# The genome sequence of the early grey, *Xylocampa areola *(Esper, 1789)

**DOI:** 10.12688/wellcomeopenres.18663.1

**Published:** 2022-12-23

**Authors:** David Lees, Douglas Boyes, Natural History Museum Genome Acquisition Lab, University of Oxford and Wytham Woods Genome Acquisition Lab, Darwin Tree of Life Barcoding collective, Wellcome Sanger Institute Tree of Life programme, Wellcome Sanger Institute Scientific Operations: DNA Pipelines collective, Tree of Life Core Informatics collective, Darwin Tree of Life Consortium

**Affiliations:** 1 Natural History Museum, London, UK; 2 UK Centre for Ecology and Hydrology, Wallingford, Oxfordshire, UK

**Keywords:** Xylocampa areola, the early grey, genome sequence, chromosomal, Lepidoptera

## Abstract

We present a genome assembly from an individual *Xylocampa areola* (the early grey; Arthropoda; Insecta; Lepidoptera; Noctuidae). The genome sequence is 565 megabases in span. Most of the assembly is scaffolded into 31 chromosomal pseudomolecules, including the assembled Z sex chromosome. The mitochondrial genome has also been assembled and is 15.5 kilobases in length. Gene annotation of this assembly on Ensembl identified 18,869 protein coding genes.

## Species taxonomy

Eukaryota; Metazoa; Ecdysozoa; Arthropoda; Hexapoda; Insecta; Pterygota; Neoptera; Endopterygota; Lepidoptera; Glossata; Ditrysia; Noctuoidea; Noctuidae; Amphipyrinae; *Xylocampa*; *Xylocampa areola* (Esper, 1789) (NCBI:txid1870430)

## Background

The early grey, *Xylocampa areola*, is a medium sized noctuid moth, intricately grey mottled with conjoined stigmata on the forewing, that starts to emerge early in the temperate moth season (usually February in the UK). The early grey is found in deciduous woodland, hedges and a wide range of other habitats, including heathland, fens, and gardens, wherever its larval foodplant, species of *Lonicera* (Caprifoliaceae) and especially *L. xylosteum*, is found. Despite its larval preference for honeysuckle, *X. areola* is not generally considered to be an important garden pest.

*Xylocampa areola* is generally common and widespread in the western Palaearctic only, from southern Scandinavia to the northern Mediterranean; but there are relatively few records of its presence in eastern Europe ( GBIF Secretariat, 2021). Populations in the UK appear to be stable or even increasing ( Conrad *et al.*, 2006; Randle *et al.*, 2019). The adult flies from February, protracted until around the end of May, with a peak in April in the UK ( Wheeler, 2022) and up to mid-June in Europe ( Kettner, 2019). Adults seek nectar at night, in the Spring particularly sallow ( *Salicaceae*) blossoms. In the Mediterranean, *X. areola* has been considered a very important Lepidoptera pollinator in the community there studied, especially of *Arbutus unedo* L., Ericaceae ( Ribas-Marquès *et al.*, 2022).

*Xylocampa* appears not to have been used in molecular phylogenies, and it would be interesting to determine its closest relatives. The sister group of the genus is apparently not known. It is currently placed in the tribe Psaphidini. The genome sequence should not only be useful in phylogeny, but in studies of potentially cryptic species. There are two DNA barcode clusters ( BOLD SYSTEMS, no date), i.e., the BINs BOLD:AAE4200 and BOLD:ABZ8138 (the latter recorded from Norway, Spain and France: Corsica, the first and more common of which already DNA barcoded for the UK), and these two discrete clusters are 1.6% divergent. There is only one other species classified in *Xylocampa*: *X. mustapha* (Oberthür, 1910) (BOLD:AEI5117), which is mainly from southern Italy, the Aegean Islands, north-west Africa, and the Middle East. The Italian population is only 2–3% divergent from BOLD:ABZ8138, and about 3.4–3.6% divergent from BOLD:AAE4200.

## Genome sequence report

**Figure 1.**
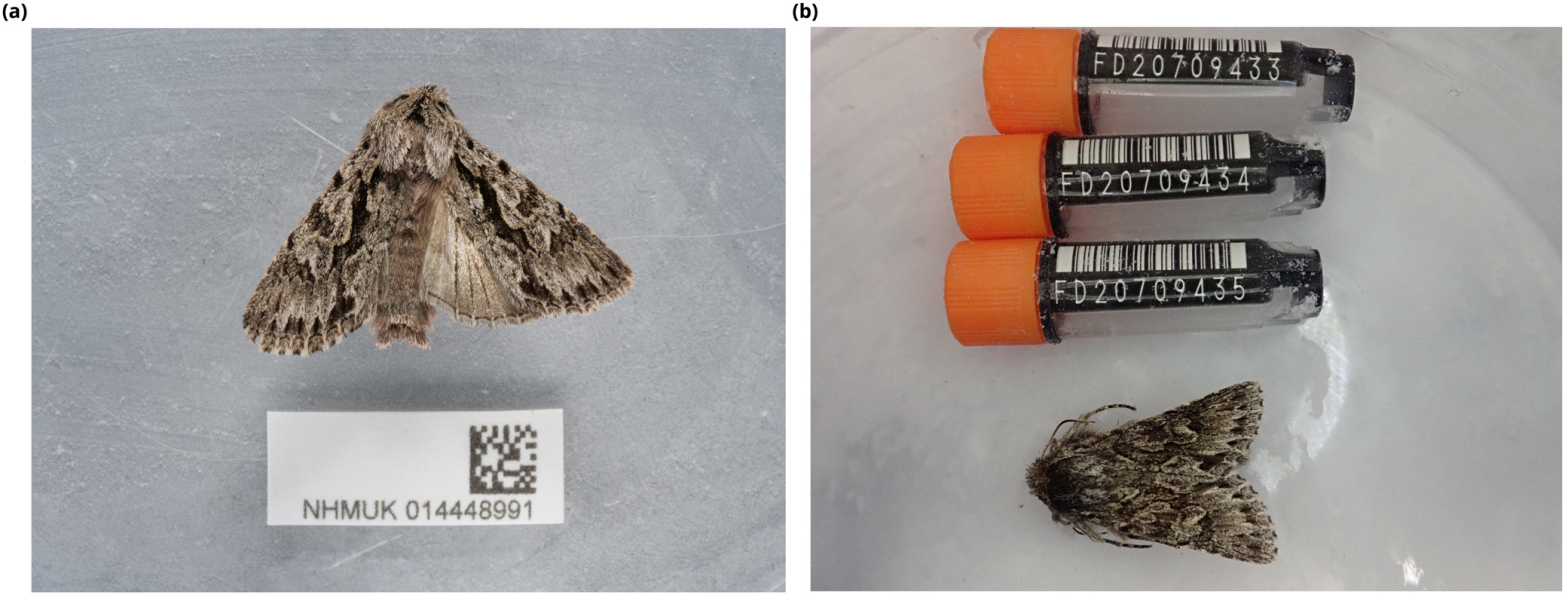
**a**) Photograph of the *X. areola* (ilXylAreo1) specimen used for genome sequencing. **b**) Photograph of the *X. areola* (ilXylAreo2) specimen used for RNA sequencing.

The genome was sequenced from an individual *X. areola* ( Figure 1) collected from High Wycombe, Buckinghamshire (NHMUK014448991, latitude 51.63, longitude –0.74). A total of 58-fold coverage in Pacific Biosciences single-molecule HiFi long reads was generated. Primary assembly contigs were scaffolded with chromosome conformation Hi-C data. Manual assembly curation corrected 8 missing/misjoins and removed 2 haplotypic duplications, reducing the scaffold number by 4.76%.

**Figure 2.**
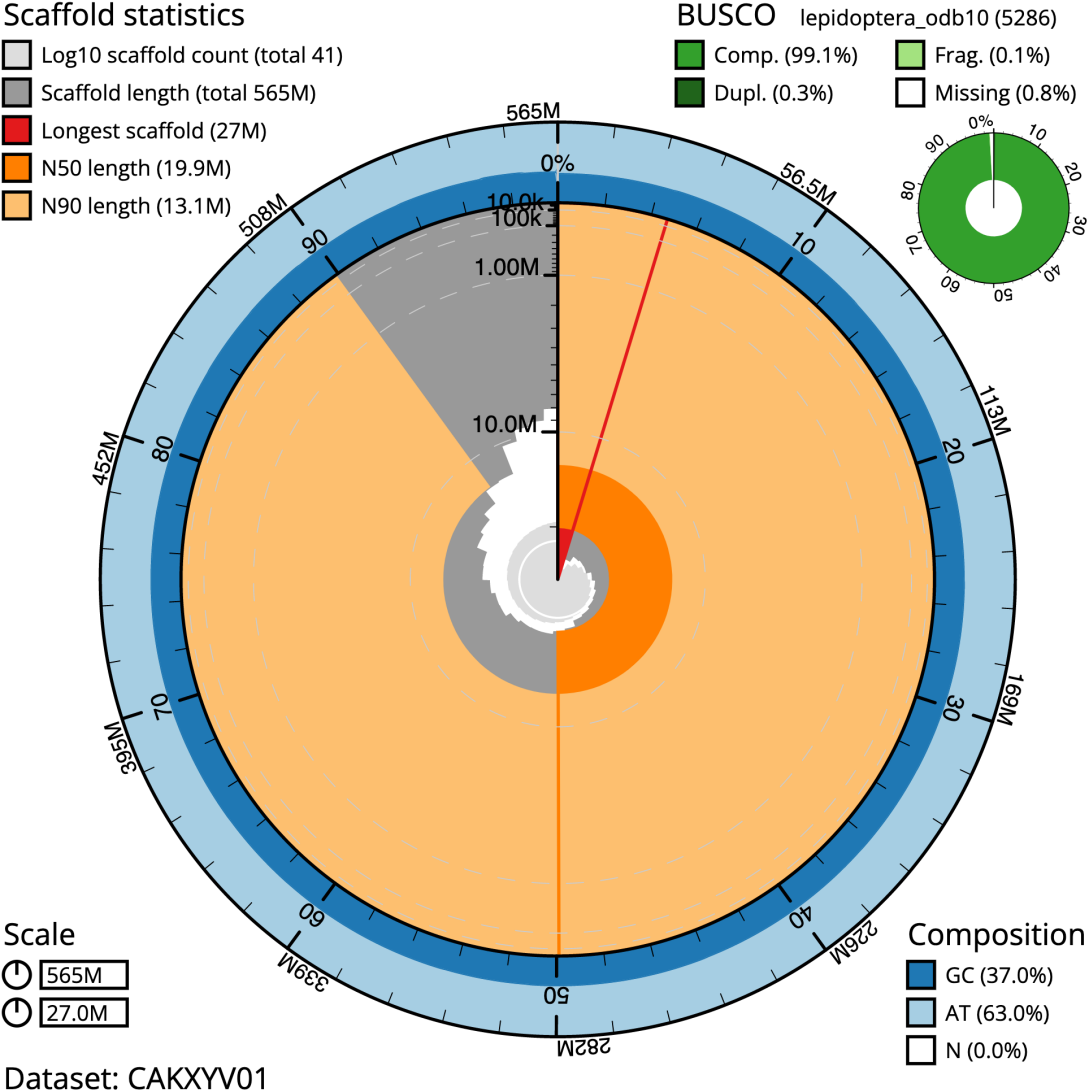
Genome assembly of *X. areola*, ilXylAreo1.1: metrics. The BlobToolKit Snailplot shows N50 metrics and BUSCO gene completeness. The main plot is divided into 1,000 size-ordered bins around the circumference with each bin representing 0.1% of the 564,568,605 bp assembly. The distribution of chromosome lengths is shown in dark grey with the plot radius scaled to the longest chromosome present in the assembly (27,003,092 bp, shown in red). Orange and pale-orange arcs show the N50 and N90 chromosome lengths (19,946,087 and 13,085,905 bp), respectively. The pale grey spiral shows the cumulative chromosome count on a log scale with white scale lines showing successive orders of magnitude. The blue and pale-blue area around the outside of the plot shows the distribution of GC, AT and N percentages in the same bins as the inner plot. A summary of complete, fragmented, duplicated and missing BUSCO genes in the lepidoptera_odb10 set is shown in the top right. An interactive version of this figure is available at https://blobtoolkit.genomehubs.org/view/ilXylAreo1.1/dataset/CAKXYV01/snail.

The final assembly has a total length of 565 Mb in 40 sequence scaffolds with a scaffold N50 of 20 Mb ( Table 1). Most (99.96%) of the assembly sequence was assigned to 31 chromosomal-level scaffolds, representing 30 autosomes and the Z sex chromosome. Chromosome-scale scaffolds confirmed by the Hi-C data are named in order of size ( Figure 2– Figure 5; Table 2). While not fully phased, the assembly deposited is of one haplotype. Contigs corresponding to the second haplotype have also been deposited.

**Table 1.** Genome data for *X. areola*, ilXylAreo1.1.

Project accession data	
Assembly identifier	ilXylAreo1.1
Species	*Xylocampa areola*
Specimen	ilXylAreo1 (DNA sequencing, Hi-C), ilXylAreo2 (RNA sequencing)
NCBI taxonomy ID	1870430
BioProject	PRJEB50748
BioSample ID	SAMEA9359445
Isolate information	Thorax tissue (PacBio), Head tissue (Hi-C), Abdomen (RNA-Seq)
Assembly metrics *	
Base pair QV	63.8 (Benchmark: ≥50)
*k*-mer completeness	100% (Benchmark: ≥95%)
BUSCO **	C:99.1%[S:98.7%,D:0.3%],F:0.1%,M:0.8%,n:5,286 (Benchmark: C ≥ 95%)
Percentage of assembly mapped to chromosomes	99.96% (Benchmark: ≥95%)
Sex chromosomes	Z chromosome assembled (Benchmark: localised homologous pairs)
Organelles	Assembled mitochondrion (Benchmark: complete single alleles)
Raw data accessions	
PacificBiosciences SEQUEL II	ERR8575387, ERR8575388
Hi-C Illumina	ERR8571674
PolyA RNA-Seq Illumina	ERR10123671
Genome assembly	
Assembly accession	GCA_935421205.1
*Accession of alternate haplotype*	GCA_935412865.1
Span (Mb)	565
Number of contigs	46
Contig N50 length (Mb)	19.6
Number of scaffolds	40
Scaffold N50 length (Mb)	19.9
Longest scaffold (Mb)	23.9
Genome annotation	
Number of protein-coding genes	18,869
Number of gene transcripts	19,064

* Assembly metric benchmarks are adapted from column VGP-2020 of “ Table 1: Proposed standards and metrics for defining genome assembly quality” from ( Rhie *et al.*, 2021). ** BUSCO scores based on the lepidoptera_odb10 BUSCO set using v5.3.2. C = complete [S = single copy, D = duplicated], F = fragmented, M = missing, n = number of orthologues in comparison. A full set of BUSCO scores is available at https://blobtoolkit.genomehubs.org/view/ilXylAreo1.1/dataset/CAKXYV01/busco.

**Figure 3.**
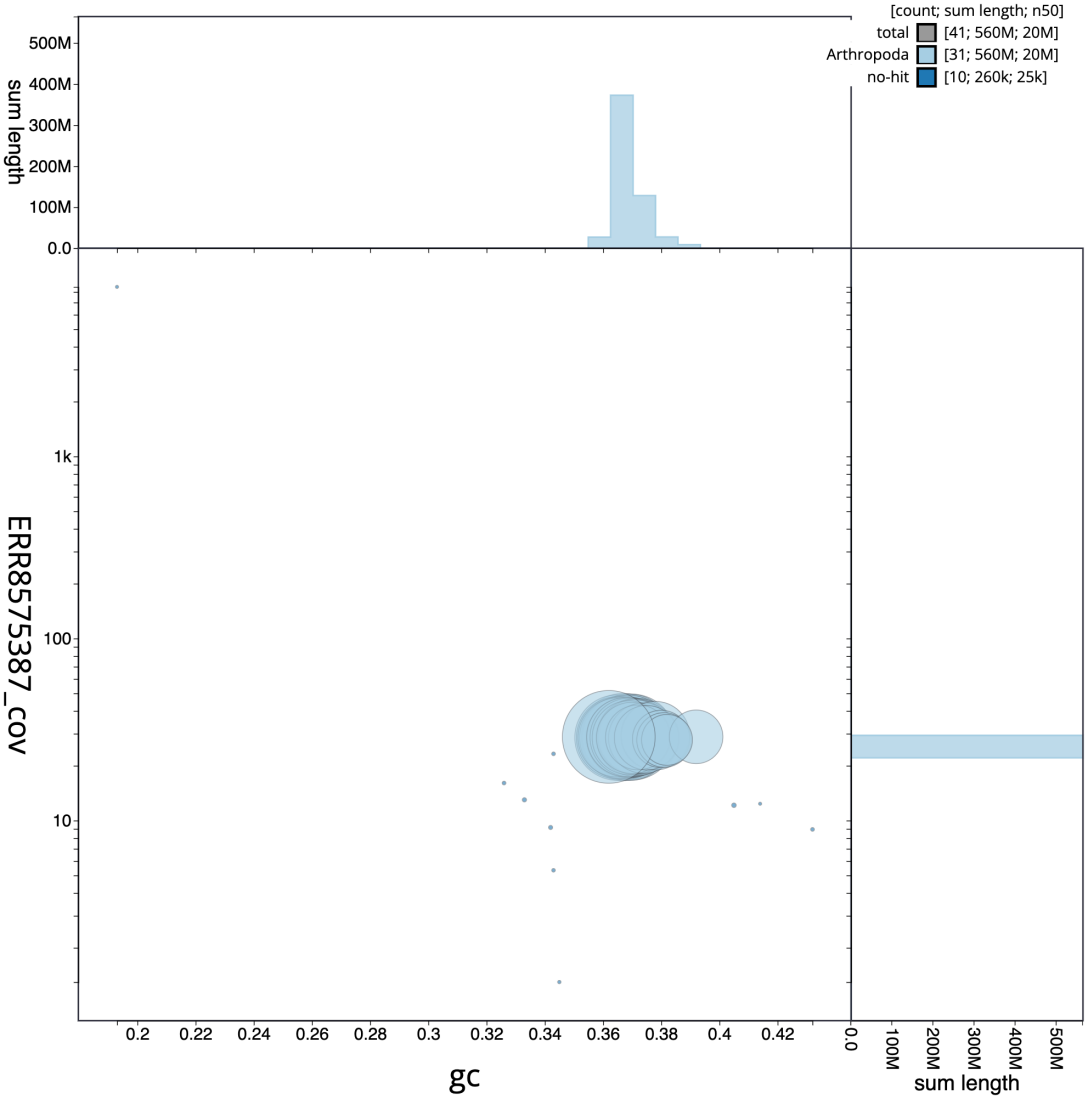
Genome assembly of *X. areola*, ilXylAreo1.1: GC coverage. BlobToolKit GC-coverage plot. Chromosomes are coloured by phylum. Circles are sized in proportion to chromosome length. Histograms show the distribution of chromosome length sum along each axis. An interactive version of this figure is available at https://blobtoolkit.genomehubs.org/view/ilXylAreo1.1/dataset/CAKXYV01/blob.

**Figure 4.**
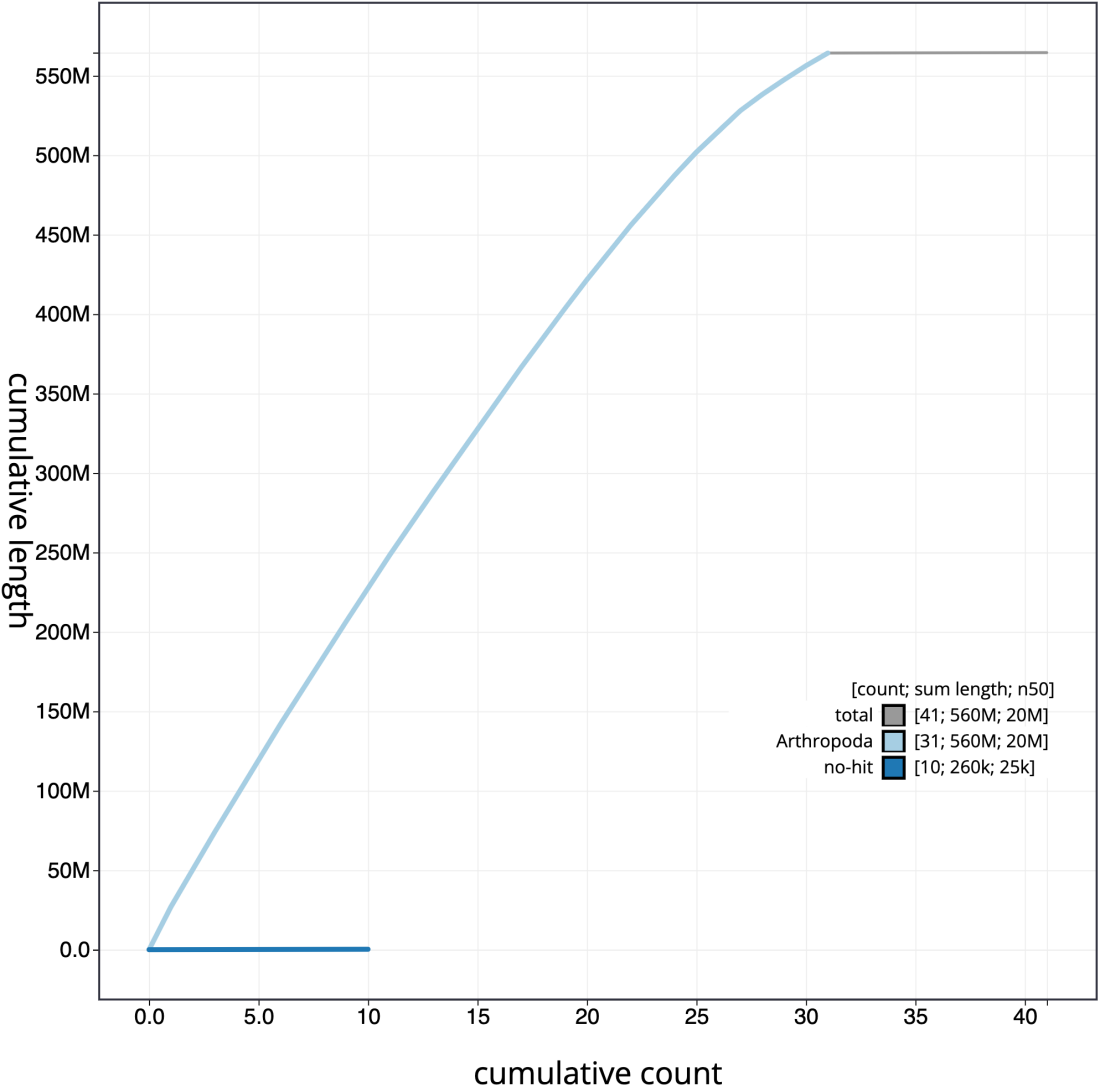
Genome assembly of *X. areola*, ilXylAreo1.1: cumulative sequence. BlobToolKit cumulative sequence plot. The grey line shows cumulative length for all chromosomes. Coloured lines show cumulative lengths of chromosomes assigned to each phylum using the buscogenes taxrule. An interactive version of this figure is available at https://blobtoolkit.genomehubs.org/view/ilXylAreo1.1/dataset/CAKXYV01/cumulative.

**Figure 5.**
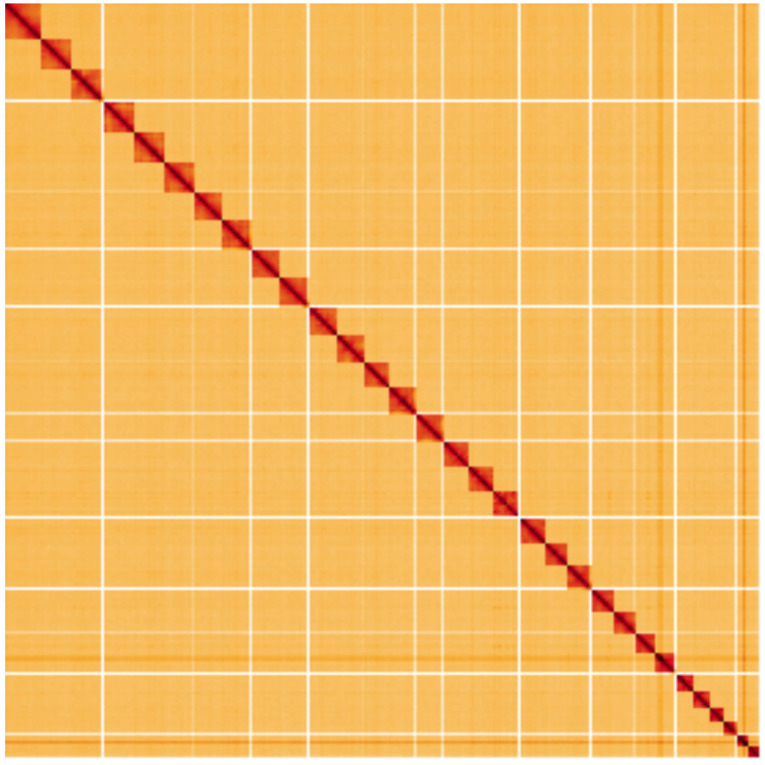
Genome assembly of *X. areola*, ilXylAreo1.1: Hi-C contact map. Hi-C contact map of the ilXylAreo1.1 assembly, visualised using HiGlass. Chromosomes are shown in order of size from left to right and top to bottom. An interactive version of this figure may be viewed at https://genome-note-higlass.tol.sanger.ac.uk/l/?d=ORLTYiKpR_y6hvw1MMLGGQ.

**Table 2.** Chromosomal pseudomolecules in the genome assembly of *X. areola*, ilXylAreo1.

INSDC accession	Chromosome	Size (Mb)	GC%
OW285200.1	1	23.89	36.7
OW285201.1	2	23.07	37
OW285202.1	3	22.89	36.5
OW285203.1	4	22.86	36.9
OW285204.1	5	22.2	36.7
OW285205.1	6	21.78	36.9
OW285206.1	7	21.49	36.9
OW285207.1	8	21.34	36.7
OW285208.1	9	21.08	36.5
OW285209.1	10	20.92	36.6
OW285210.1	11	20.2	36.9
OW285211.1	12	19.95	36.8
OW285212.1	13	19.6	36.5
OW285213.1	14	19.58	36.8
OW285214.1	15	19.54	36.8
OW285215.1	16	19.21	36.9
OW285216.1	17	18.55	37.1
OW285217.1	18	18.5	37.2
OW285218.1	19	18.03	37.1
OW285219.1	20	17.17	37.1
OW285220.1	21	17.1	36.9
OW285221.1	22	15.88	37
OW285222.1	23	15.48	37.4
OW285223.1	24	14.65	37.8
OW285224.1	25	13.09	37.2
OW285225.1	26	12.97	37.5
OW285226.1	27	10.21	38
OW285227.1	28	9.24	38.1
OW285228.1	29	8.89	39.2
OW285229.1	30	7.98	38.2
OW285199.1	Z	27	36.2
OW285230.1	MT	0.02	19.4
-	-	0.24	36.6

The assembly has a BUSCO v5.3.2 ( Manni *et al.*, 2021) completeness of 99.1% (single, 98.7%, duplicated 0.3%) using the lepidoptera_odb10 reference set.

## Genome annotation report

The *Xylocampa areola* genome assembly (GCA_935421205.1) was annotated using BRAKER2 ( Table 1; https://rapid.ensembl.org/Xylocampa_areola_GCA_935421205.1/Info/Index). The resulting annotation includes 19,064 transcribed mRNAs from 18,869 protein-coding genes.

## Methods

### Sample acquisition and nucleic acid extraction

An unsexed individual *X. areola* (ilXylAreo1) was collected and identified by David Lees (Natural History Museum, London) from High Wycombe, Buckinghamshire, UK (latitude 51.63, longitude –0.74). The sample was preserved by dry freezing at –80°C.

A second unsexed *X. areola* (ilXylAreo2) was collected in Wytham Woods, Berkshire, UK (latitude 51.77, longitude –1.34) by Douglas Boyes (University of Oxford). The specimen was identified by Douglas Boyes and snap-frozen on dry ice.

DNA was extracted at the Tree of Life laboratory, Wellcome Sanger Institute. The ilXylAreo1 sample was weighed and dissected on dry ice with head tissue set aside for Hi-C sequencing. The whole body tissue was disrupted using a Nippi Powermasher fitted with a BioMasher pestle. High molecular weight (HMW) DNA was extracted using the Qiagen MagAttract HMW DNA extraction kit. Low molecular weight DNA was removed from a 20 ng aliquot of extracted DNA using 0.8X AMpure XP purification kit. HMW DNA was sheared into an average fragment size of 12–20 kb in a Megaruptor 3 system with speed setting 30. Sheared DNA was purified by solid-phase reversible immobilisation using AMPure PB beads with a 1.8X ratio of beads to sample to remove the shorter fragments and concentrate the DNA sample. The concentration of the sheared and purified DNA was assessed using a Nanodrop spectrophotometer and Qubit Fluorometer and Qubit dsDNA High Sensitivity Assay kit. Fragment size distribution was evaluated by running the sample on the FemtoPulse system.

RNA was extracted from the abdomen tissue of ilXylAreo2 in the Tree of Life Laboratory at the WSI using TRIzol, according to the manufacturer’s instructions. RNA was then eluted in 50 μl RNAse-free water and its concentration assessed using a Nanodrop spectrophotometer and Qubit Fluorometer using the Qubit RNA Broad-Range (BR) Assay kit. Analysis of the integrity of the RNA was done using Agilent RNA 6000 Pico Kit and Eukaryotic Total RNA assay.

### Sequencing

Pacific Biosciences HiFi circular consensus libraries were constructed according to the manufacturers’ instructions. Poly(A) RNA-Seq libraries were constructed using the NEB Ultra II RNA Library Prep kit. DNA and RNA sequencing were performed by the Scientific Operations core at the WSI on Pacific Biosciences SEQUEL II (HiFi) and Illumina NovaSeq 6000 (RNA-Seq) instruments. Hi-C data were also generated from head tissue of ilXylAreo1 using the Arima v2 kit and sequenced on the Illumina NovaSeq 6000 instrument.

### Genome assembly

Assembly was carried out with Hifiasm ( Cheng *et al.*, 2021) and haplotypic duplication was identified and removed with purge_dups ( Guan *et al.*, 2020). The assembly was scaffolded with Hi-C data ( Rao *et al.*, 2014) using YaHS ( Zhou *et al.*, 2022). The assembly was checked for contamination as described previously ( Howe *et al.*, 2021). Manual curation ( Howe *et al.*, 2021) was performed using HiGlass ( Kerpedjiev *et al.*, 2018) and Pretext ( Harry, 2022). The mitochondrial genome was assembled using MitoHiFi ( Uliano-Silva *et al.*, 2021), which performed annotation using MitoFinder ( Allio *et al.*, 2020). The genome was analysed and BUSCO scores were generated within the BlobToolKit environment ( Challis *et al.*, 2020). Table 3 contains a list of all software tool versions used, where appropriate.

**Table 3.** Software tools and versions used.

Software tool	Version	Source
BlobToolKit	3.4.0	Challis *et al.*, 2020
Hifiasm	0.16.1-r375	Cheng *et al.*, 2021
HiGlass	1.11.6	Kerpedjiev *et al.*, 2018
PretextView	0.2.x	Harry, 2022
purge_dups	1.2.3	Guan *et al.*, 2020
MitoHiFi	2.0	Uliano-Silva *et al.*, 2021
YaHS	yahs-1.1.91eebc2	Zhou *et al.*, 2022

### Genome annotation

BRAKER2 ( Brůna *et al.*, 2021) was used to generate annotation for the *Xylocampa areola* assembly (GCA_935421205.1). Annotation was created primarily through alignment of transcriptomic data to the genome, with gap filling via protein to-genome alignments of a select set of proteins from UniProt ( UniProt Consortium, 2019).

## Data Availability

European Nucleotide Archive: *Xylocampa areola*. Accession number PRJEB50748; https://identifiers.org/ena.embl/PRJEB50748 ( Wellcome Sanger Institute, 2022). The genome sequence is released openly for reuse. The *Xylocampa areola* genome sequencing initiative is part of the Darwin Tree of Life (DToL) project. All raw sequence data and the assembly have been deposited in INSDC databases. Raw data and assembly accession identifiers are reported in Table 1.

## Author information

Members of the Natural History Museum Genome Acquisition Lab are listed here: https://doi.org/10.5281/zenodo.4790042.

Members of the University of Oxford and Wytham Woods Genome Acquisition Lab are listed here: https://doi.org/10.5281/zenodo.4789928.

Members of the Darwin Tree of Life Barcoding collective are listed here: https://doi.org/10.5281/zenodo.4893703.

Members of the Wellcome Sanger Institute Tree of Life programme are listed here: https://doi.org/10.5281/zenodo.4783585.

Members of Wellcome Sanger Institute Scientific Operations: DNA Pipelines collective are listed here: https://doi.org/10.5281/zenodo.4790455.

Members of the Tree of Life Core Informatics collective are listed here: https://doi.org/10.5281/zenodo.5013541.

Members of the Darwin Tree of Life Consortium are listed here: https://doi.org/10.5281/zenodo.4783558.
