# Supplementary material for: The genome sequence of the early grey, Xylocampa areola (Esper, 1789)
Source: Wellcome Open Res. 2022 Dec 23;7:312. [Version 1] doi: 10.12688/wellcomeopenres.18663.1 (PMC10687390; doi:10.12688/wellcomeopenres.18663.1)
Supplement: Peer review history

# Reviewer report: Reviewer response for version 1

**DOI:** 10.21956/wellcomeopenres.20696.r68071

**Published:** 2023-11-29

**Authors:** Fernando Seixas

**Affiliations:** 1 Department of Organismic and Evolutionary Biology, Harvard University, Cambridge, Massachusetts, USA

The authors assembled the genome of one Xylocampa areola individual, using PacBio and Hi-C data. This resulted in a complete (as attested by the BUSCO score) and highly contiguous (i.e. chromosome-level) assembly. I found the writing very clear, and the authors always provide complete information to understand their work. The one point that could be clarified is in the "Genome annotation" section, where the authors say "Annotation was created ***primarily*** through alignment of transcriptomic data". The ***primarily*** in that sentence makes me believe that other information was used (I could presume homology-based and/or de novo annotation) so it would be helpful if the authors could explain what other sources of evidence were used. Otherwise, I have no other remarks.

Are sufficient details of methods and materials provided to allow replication by others?

Yes

Is the rationale for creating the dataset(s) clearly described?

Yes

Are the datasets clearly presented in a useable and accessible format?

Yes

Are the protocols appropriate and is the work technically sound?

Yes

Reviewer Expertise:

Evolutonary Biology

I confirm that I have read this submission and believe that I have an appropriate level of expertise to confirm that it is of an acceptable scientific standard.

# Reviewer report: Reviewer response for version 1

**DOI:** 10.21956/wellcomeopenres.20696.r69790

**Published:** 2023-11-27

**Authors:** Lars Höök

**Affiliations:** 1 Department of Ecology and Genetics, Uppsala University, Uppsala, Sweden

In this data note, the authors present the genome assembly of Xylocampa areola, the early grey moth. The assembly consist of 31 chromosome scaffolds which is common for a Lepidoptera species.

 The methods are well described and follow current standards for generating high quality genome assemblies. The genome assembly, as descibed in the note, is of high quality and is therefore a valuable resource for studies of evolution and phylogenetics.

 The relevant information describing the assembly is clearly presented and the genome data is made available at ENA.

Are sufficient details of methods and materials provided to allow replication by others?

Yes

Is the rationale for creating the dataset(s) clearly described?

Yes

Are the datasets clearly presented in a useable and accessible format?

Yes

Are the protocols appropriate and is the work technically sound?

Yes

Reviewer Expertise:

Evolutionary genetics

I confirm that I have read this submission and believe that I have an appropriate level of expertise to confirm that it is of an acceptable scientific standard.
